# Energy–Accuracy Aware Finger Gesture Recognition for Wearable IoT Devices

**DOI:** 10.3390/s22134801

**Published:** 2022-06-25

**Authors:** Woosoon Jung, Hyung Gyu Lee

**Affiliations:** 1 Department of Computer and Information Engineering, Daegu University, Gyeongsan-si 38453, Korea; quado.jung@gmail.com; 2 Department of Software, Duksung Women’s University, Seoul 01369, Korea

**Keywords:** MLP, gesture recognition, flex sensor, model search, neural network

## Abstract

Wearable Internet of Things (IoT) devices can be used efficiently for gesture recognition applications. The nature of these applications requires high recognition accuracy with low energy consumption, which is not easy to solve at the same time. In this paper, we design a finger gesture recognition system using a wearable IoT device. The proposed recognition system uses a light-weight multi-layer perceptron (MLP) classifier which can be implemented even on a low-end micro controller unit (MCU), with a 2-axes flex sensor. To achieve high recognition accuracy with low energy consumption, we first design a framework for the finger gesture recognition system including its components, followed by system-level performance and energy models. Then, we analyze system-level accuracy and energy optimization issues, and explore the numerous design choices to finally achieve energy–accuracy aware finger gesture recognition, targeting four commonly used low-end MCUs. Our extensive simulation and measurements using prototypes demonstrate that the proposed design achieves up to 95.5% recognition accuracy with energy consumption under 2.74 mJ per gesture on a low-end embedded wearable IoT device. We also provide the Pareto-optimal designs among a total of 159 design choices to achieve energy–accuracy aware design points under given energy or accuracy constraints.

## 1. Introduction

Gesture recognition is among the popular issues for human–machine interface applications. In particular, hands are the parts that can move most accurately with relatively little energy, compared to other body parts. Thus, hand gesture recognition is used as an efficient interface for human–computer interaction (HCI) [1,2,3,4,5,6,7,8]. Traditionally, vision-based gesture recognition received much attention since it avoid the need to wear any tools or equipment on the body [1,2,6]. However, it is also known that the performance of vision-based gesture recognition is highly dependent on camera setup such as the angle to the object, the size of the image and the intensity of illumination [9]. In addition, high computation requirements and power consumption are needed to process and analyze multiple images in real time. Thus, it may not be feasible to implement vision-based gesture recognition applications on low-end embedded devices.

An alternative method of implementing gesture recognition is to use wearable sensors such as inertial measurement units (IMU), electromyography (EMG) sensors, flex sensors, and pressure sensors [3,8,10,11,12,13]. Unlike vision-based approaches, a wearable sensor-based approach is not only less sensitive to the perceived environments but also generates relatively small amounts of data with affordable (or even higher) recognition accuracy. In addition, this approach can recognize minimal body movements including small finger gestures. Most of all, its computation and power requirements may be less than vision-based approaches. In that sense, a wearable sensor-based approach is more suitable for gesture recognition than a vision-based approach if we are targeting low-end wearable IoT devices.

Among various wearable sensors such as IMUs, EMG, and flex sensors, we focus on using a state-of-the-art flex sensor [14] which can measure bi-directionally in 2 axes of bending with a single sensor. This sensor is suitable for being implemented in low-end embedded devices because it provides low-power, drift-free, and path-independent sensing with high accuracy. In addition, the sensor is made from silicon, which is good for wearable implementations. 

In this paper, we design a light-weight finger gesture recognition system that can be implemented in low-end embedded devices using a single flex sensor. To this end, we first design a framework for a finger gesture recognition system that recognizes 17 finger gestures. The framework consists of data collection, preprocessing filters, and a light-weight multi-layer perceptron (MLP)-based classifier. Then, we construct performance and energy models to find optimal design choices efficiently. We analyze and discuss the energy–accuracy aware system-level design issues, and explore the design choices of finger gesture recognition by considering computation requirements/memory resource targeting for four types of low-end micro controller units (MCUs). Finally, the functionality and feasibility of the proposed work are verified by implementing prototypes. The contributions of this paper are summarized as follows:

- Provide the full design for a finger gesture recognition system using a single flex sensor.
- Explore the design choices of a finger gesture recognition system in terms of performance, accuracy, and energy consumption using the conducted performance and energy consumption models.
- Demonstrate the functionality and feasibility of the proposed designs by implementing the prototypes using four commonly used low-end embedded MCUs.
- Show the energy–accuracy aware design which achieves up to 95.5% accuracy with an energy consumption of 2.74 mJ per gesture.
- Provide the energy–accuracy aware Pareto-optimal designs among a total of 159 design choices to find energy–accuracy aware design points under given energy or accuracy constraints.

The rest of this paper is organized as follows. The backgrounds are described in Section 2. In Section 3, the framework and component-level design for the finger gesture recognition system are described, while Section 4 discusses energy–accuracy aware design optimization. Finally, Section 5 demonstrates the experiment results, followed by the conclusion in Section 6.

## 2. Backgrounds 

This section describes the backgrounds of this work which consists of the existing work related to gesture recognition and the basics of the flex sensor used in this work.

### 2.1. Related Work

An IMU sensor which embeds micro electro mechanical systems (MEMS) accelerometers, gyroscopes, and magnetometers was popularly used because it can capture the wide range of body movements. An IMU sensor can even be attached to a cane to detect falls in the elderly [10]. However, IMUs generally require high filtering resources because raw data contain a lot of noise and drifts [4]. In addition, a high sampling rate (higher than a few kilo samples per second) requirement for recognizing delicate movements and high recognition accuracy are major concerns for implementing on low-end embedded devices [15].

EMG sensors are used for body movement recognition as well. Instead of directly measuring the physical movements of the body, the sensor alternatively measures the biomedical signals using specially made probes attached to the skin surface. EMG sensors can detect the very fine movements of the body that cannot be detected by physical movement measuring sensors alone [3,16,17]. However, the acquired biomedical signals vary for different people even with the same movement and are noise sensitive depending on the condition of the skin surface even for the same person [18].

Conventional flex sensors based on conductive ink, fiber-optic, or conductive fabric technologies are used for various wearable IoT applications such as embedded device-based health care [19], sign language recognition [20,21], and posture correction [22]. Multiple sensors are attached to each joint of the body, and the measured bending information is used for recognizing the body movement. This method provides a low-cost and low-energy solution that can be easily implemented in low-end embedded devices. However, the recognized body activity is generally simple and must use multiple sensors to detect complex body movements. Recently, an advanced flex sensor that can measure two axes of bi-directional bending with a single sensor was developed [14]. The sensor embeds a low-power integrated analog front and generates digital angular data in degree. We use this advanced flex sensor for finger gesture recognition in this paper. Thus, the details on this flex sensor will be explained in Section 2.2.

In general, data collected from the wearable flex sensor for body movement recognition requires time-domain data analysis using machine learning (ML) techniques such as dynamic time warping (DTW) [20], hidden Markov models (HMMs) [21], recurrent neural networks (RNNs), and long short-term memory (LSTM). Although these techniques support relatively high recognition accuracy for time-series data, it is questionable whether these techniques can be efficiently implemented in a low-end wearable device [7,23] because of the not trivial size of memory requests. Since the data used in HCI applications generally have a small number of dimensions compared to the images, a simple MLP technique can be a sufficient solution if it satisfies the desired performance and accuracy. Therefore, this paper focuses on using an MLP technique where the computation requirements (processing time) are simply proportional to the size of MLP model. The optimal MLP structure was determined in terms of model size, accuracy, and energy consumption in this paper.

### 2.2. Basics of Flex Sensors

**Figure 1:**
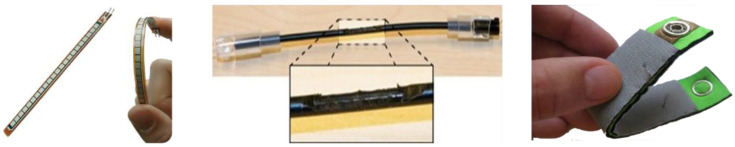
Types of flex sensors (from left, conductive ink, fiber-optic, and conductive fabric based).

Flex sensors measure the amount of bending or deflection. There are three types of commonly used flex sensors, as shown in Figure 1. Depending on the material, the sensor is categorized as conductive ink, fiber-optic, or conductive fabric. The operating principle of the sensors utilizes a phenomenon where the electrical properties of a material used in the sensor change when the flex sensor is physically bending. Depending on the type of flex sensor, the maximum bending angle, durability, and stability of the measured value appear differently. For example, sensors made with conductive ink are widely used due to low cost, but accuracy is relatively low, and calibration or filtering is required because the measured values vary slightly depending on the measurement environment such as temperature and humidity. In addition, the physical length of the sensor is fixed without elasticity, which limits the wearability of the sensor. 

Sensors made with an optical fiber support high accuracy and high durability. However, a pair of a light source and a detector is required, and only unidirectional sensing is possible [24]. Conductive fabric/polymers can be used for wearable applications due to the elasticity of the sensor compared to other technologies. The cost of these sensors is relatively high, compared with other types of sensors, and these sensors respond to pressure as well as bending, making it difficult to maintain high accuracy. Most of all, conventional flex sensors can measure one axis of bending. Thus, multiple sensors must be used to measure complex movements [13].

The advanced flex sensor introduced in the previous subsection is made with a silicone elastomer layered with a conductive and non-conductive material. This sensor not only measures the bending degree of two axes stably with a single sensor, but also has the advantage of being flexible and stretchable with silicon material. As mentioned, this sensor is not a simple variable resistor type but a sensor module that embeds a low-power integrated analog front, resulting much less noise over time compared with the other sensors. In addition, it generates digital data through an inter-integrated circuit (I^2^C) standard communication interface. This means that power-hungry analog-to-digital converters (ADCs) are not necessary, which is good for wearable IoT devices.

**Figure 2:**
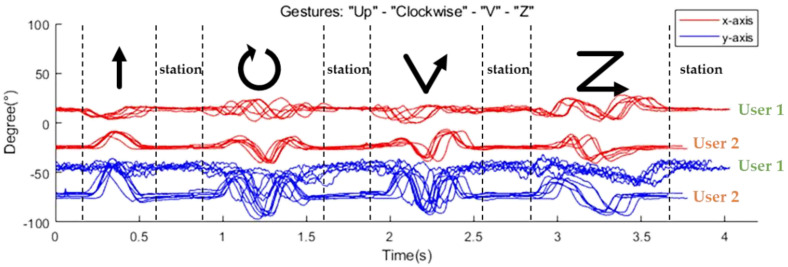
Example outputs of the flex sensor (four types of gestures from two users).

Figure 2 shows the collected sample data from two users, repeating several gestures with their index fingers, where a single flex sensor is attached. The measured values indicate the angle changes according to the movement of the finger. Although there are slight deviations in the measured values of each repeated gesture, we observe specific patterns for each gesture regardless of the users. These patterns appear differently depending on the type of gesture. We also note that the duration of a single gesture—the number of sample data related to the gesture—varies depending on the type of the gesture and user. The duration of a single gesture also varies depending on the time even for the same gesture by the same person. Therefore, gesture recognition should be appropriately designed in consideration of these variations.

## 3. Designing the Finger Gesture Recognition System

This section mainly describes the design for a light-weight finger gesture recognition system using a wearable flex sensor, implemented in low-end wearable devices. To this end, the system-level design including its framework is proposed. Then, the component-level design consisting of designing preprocessing filters and an MLP-based classifier is described.

### 3.1. System Architecture

**Figure 3:**
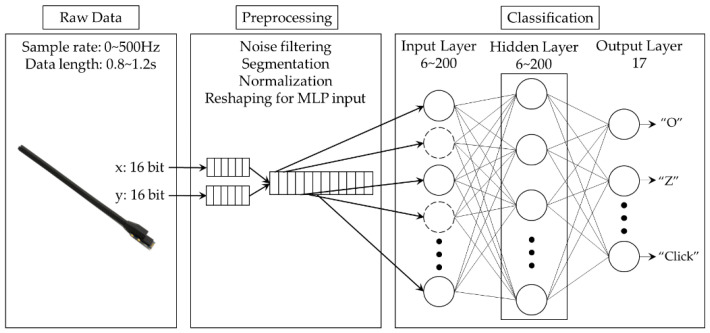
The framework for the proposed finger gesture recognition system.

Figure 3 shows the framework for the proposed finger gesture recognition system. The system simply consists of three parts: raw data collection, preprocessing, and classification. The first step for finger gesture recognition is to collect motion data generated from a 2-axes flex sensor. The flex sensor attached to the index finger generates a series of 32-bit sample data. One set of sample data represents the X-axis (16 bits) and Y-axis (16 bits) bending degrees of the index finger at the moment of sampling. The flex sensor can operate at a sampling rate of up to 500 Hz. In this work, we set the maximum sampling frequency to 100 Hz, which is sufficient for finger gesture recognition applications.

Raw data collected from the flex sensor can be directly used as an input to the gesture classifier. However, in general, the raw data may include lots of measurement noise and there are non-negligible deviations in the raw data collected even for the same gestures depending on the time and user, as shown in Figure 2. Additionally, the group of data sent to the classifier for gesture recognition should not be mixed with other sample data related to past or future gestures. Without resolving these problems prior to classification, recognition accuracy can be degraded while the computation requirements and energy consumption during the classification process can be increased significantly. For this reason, we design preprocessing filters which will be described in detail in Section 3.2.

Finally, the preprocessed group of data is sent to the classifier for recognizing the gesture among predefined ones. The main purpose of this study is to design and implement a gesture recognition system with high accuracy that can be implemented even on a low-end embedded device which operates with a limited energy resource such as a tiny battery or via energy harvesting. To this end, we design a light-weight MLP-based classifier to decrease computation requirements and energy consumption to as low as possible. The design and optimization of this MLP-based classifier will be explained in Section 3.3.

### 3.2. Designing Preprocessing Filters

**Figure 4:**
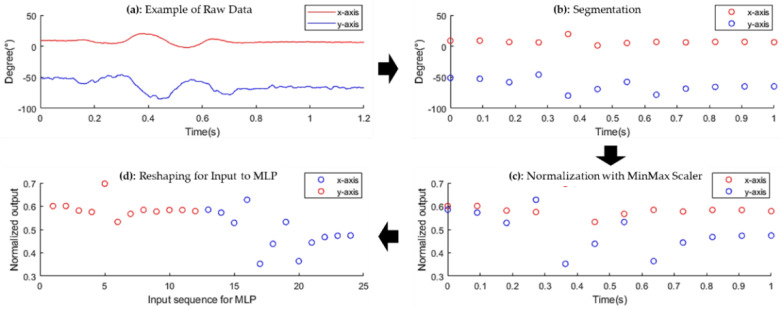
Preprocessing flow of the raw data collected.

In this section, we design preprocessing filters that convert the shape of data, as shown in Figure 4 by applying a noise filter, a segmentation filter, a normalization filter, and a reshape filter in order.

**Noise filter:** No matter how well the sensor circuit is designed, it is unavoidable that the raw data contain a lot of noise during data collection from the sensor, as shown in Figure 4a. Noise is generated in a random and non-uniform pattern, which makes detecting the unique pattern of each gesture even more difficult, and finally requires more computation. To minimize the effect of noise, we use an infinite impulse response (IIR), where the input signal and output signal are applied recursively to perform filtering. This IIR filter is more suitable for our work than a finite impulse response (FIR) filter because of its low implementation cost and low latency.

**Data segmentation filter:** The segmentation filter first separates a group of data, only related to a single gesture among continuously collected data from the sensor. To design this segmentation function, we investigate an average rate of change in sampled data to indicate the start and end of collecting a group of data only related to a single gesture, assuming that the finger is not moving for a certain amount of the time before and after each gesture. The average rate of change can be simply calculated at the same time as executing the noise filter so that the overhead for calculating the average rate of change is minimized. Starting from a steady state, the collection is started if the average rate of change is over the predefined threshold, and the collection is stopped if the average rate of change is under the predefined threshold as well. We reasonably set this threshold empirically after intensive experiments. 

The second role of the segmentation filter is to change the variable number of sampled data for a single gesture to the fixed number. As mentioned, the number of sample data grouped into a single gesture varies depending on gesture type, user, and time of trial. If this number varies, it is difficult to apply a simple MLP-based classifier. To resolve this issue, we interpolate the data if the number of data is smaller than the predefined number while we reduce the number of data by applying a smoothing function in the opposite case, so that the number of sampled data to the classifier is fixed with the predefined one, as shown in Figure 4b. Since the number of data to be sent to the classifier for a single gesture recognition is also tightly coupled with setting the sample rate of the flex sensor and designing a classifier as well, we discuss this issue in Section 4, separately.

**Normalization and Reshaping:** Normalization is an efficient method for an MLP-based classifier to increase recognition accuracy while reducing the computation requirements by adjusting the amplitude of data. We use a MinMax scaler, which normalizes the amplitude of data based on maximum and minimum values among the whole set of data, as shown in Figure 4c. Note that minimum and maximum values of the data are determined during the segmentation, the additional overhead of this process is almost negligible. The last process before sending the data to the classifier is reshaping the output of the sensor to fit the input of the MLP with a predefined size. Since the output of sensor data is 16 bits from the X-axis and 16 bits from the Y-axis, it is converted from 2D array to 1D array data, as shown in Figure 4d. This process is simple, with almost no computational overhead for this process if this process is performed with the normalization process.

### 3.3. Designing an MLP-Based Classifier

For recognizing hand gestures, we design a simple MLP-based classifier but support high recognition accuracy using minimal resources. This section only describes a classifier design and component-level optimization issue while system-level optimization issues will be discussed in Section 4.

**Input Layer:** In designing the input layer of an MLP-based classifier, the number of nodes is mainly determined by the size of the input data set. In our design, since the segmentation filter determines the size of the input data set with a predefined number, the number of nodes in the input layer is also designed to have the same number with the predefined one in the segmentation filter.

**Hidden Layer:** Determining the number of hidden layers and the number of nodes for each hidden layer is a main design issue because they are directly related to the amount of computing, memory space, and energy consumption, in addition to recognition accuracy. Huge design choices include selecting a proper structure for the hidden layer. In this work, the amount of data generated by the flex sensor is smaller compared with that of image processing. Thus, the number of hidden layers we consider is limited to a single or a double hidden layer. To find the best solution, we intensively explore the design choices of the MLP-based classifier by changing the number of nodes used for each layer in terms of recognition accuracy, energy consumption, and the feasibility of implementation considering the performance and memory size targeting low-end embedded devices. Each node in the hidden layer uses a rectified linear unit (ReLu) activation function. For each explored MLP model, we perform an independent training and testing process. The exploration in detail will be described with system-level optimization in Section 4, while the results will be described in Section 5.

**Output Layer:** The number of nodes in the output layer is generally determined by the number of recognized gestures. In this work, the number of gestures is set to 17. Thus, we design the output layer to have 17 nodes. Each node in the output layer uses a Softmax activation function to generate a probability value for each gesture so that the gesture with the highest probability is selected as the final result.

## 4. Energy–Accuracy Aware Design Optimization

Based on the design described in Section 3, this section analyzes the implementation issues of energy–accuracy aware system-level optimization targeting low-end embedded devices. We first analyze the practical issues of designing an entire system focusing on performance and power management. Then, we build performance and energy estimation models to find the energy–accuracy trade-offs. Finally, energy–accuracy aware system-level design optimization is described. 

### 4.1. Performance (Timing) Estimation Models

**Figure 5:**
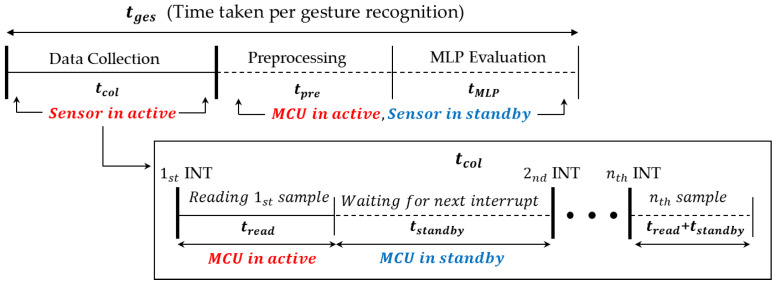
The routine (loop) that performs classification.

In terms of the design components, the proposed system consists of data collection, preprocessing filters, and an MLP-based classifier. At the same time, in terms of hardware components, the system mainly consists of a flex sensor and an MCU board. Thus, management of these hardware components is a practical issue of the implementation. For example, activation/deactivation scheduling of the MCU and the sensor module is tightly coupled with the performance and energy consumption of the system. The MCU can be in a standby state synchronized with the operating frequency of the sensor. When the preprocessing and MLP classification tasks are executed in the MCU, the sensor can be entered into a standby state to minimize the power consumption of the sensor. To address these issues, we first build timing models of gesture recognition, as shown in Figure 5. Table 1 describes the parameters used in our timing models.

The time taken per single gesture recognition, $t_{ges}$, is defined as the sum of the time for executing data collection, $t_{col}$, which is equal to the duration of a gesture, the time for preprocessing, $t_{pre}$, and the time for MLP classification, $t_{MLP}$. Depending on the user and the type of gesture, $t_{col}$ varies from 0.8 s to 1.2 s based on our experiences. $t_{pre}$ and $t_{MLP}$ vary from 33 μs to 1727 μs, and 284 μs to 3360 μs, respectively, depending on the number of sensor data, the size of MLP models, and the type of MCUs.

Looking at the data collection process which accounts for most of the time spent on gesture recognition, the MCU repeats the sensor data read with the sampling frequency $f_{s}$. At each period of read, the MCU reads a single data set, and then transits back to the standby state, waiting for the next interruption from the sensor. The time for reading a single set of data is defined as $t_{read}$, and the time spent in the standby state is defined as $t_{standby}$. In our experiments, $t_{read}$ is measured as 269 μs, which is determined by the I^2^C configuration when running at 400 KHz. Note that the sensor is always in the active state during $t_{col}$, while it is in the standby state during $t_{pre}$ and $t_{MLP}$. Since $t_{col}$ varies only with the type of gesture and user, and not with the design parameters, the number of sampled data per gesture to be recognized, N, is calculated as:

(1) $$N=t_{col}*f_{s}$$

When estimating $t_{pre},$ since we expect that it is proportional to N, we model it as a simple function of N. We also expect that $t_{MLP}$ may be proportional to N because N determines the number of nodes in the input layer. However, since N varies depending on the gesture and user, we change N into $N^{\prime }$, which is a fixed number in the segmentation process. In addition to $N^{\prime }$, $t_{MLP}$ is also tightly coupled with the size of MLP parameters, $N_{MLP}$. Thus, we model $t_{MLP}$ as a function of $N^{\prime }$ and $N_{MLP}$. Based on the scenario described above, $t_{ges}$ can be estimated as follows:

(2) $$t_{ges}=N*\frac{1}{f_{s}}+t_{pre}\left(N\right)+t_{MLP}\left(N^{\prime },N_{MLP}\right)$$

Since our design considers $N^{\prime }$ as close to N as possible, $t_{ges}$ is mainly affected by $f_{s}$ and $N_{MLP}$ because N is, in turn, determined by $f_{s}$, as shown in Equation (1). We find $t_{pre}\left(N\right)$ and $t_{MLP}\left(N^{\prime },N_{MLP}\right)$ from the extensive measurements using several low-end MCU prototypes which will be explained in Section 5.

### 4.2. Energy Estimation Models

**Figure 6:**
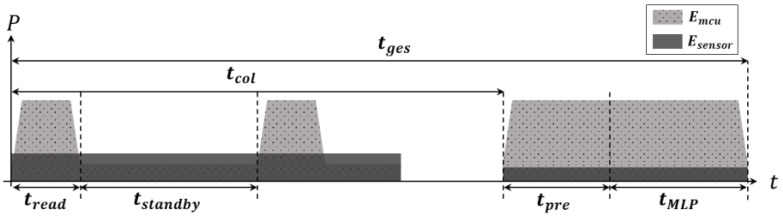
Visualized energy consumption over the time.

Figure 6 visualizes the power consumption of two main hardware components during $t_{col}$, $t_{pre}$ and $t_{MLP}$. Considering the complexity of power management, our design only uses two power states—active and standby—for both the MCU and the sensor.

The energy consumption per single gesture recognition, $E_{ges}$, is defined as the sum of the energy consumption in the MCU, $E_{mcu}$, and the energy consumption in the sensor, $E_{sensor}$. The energy consumption of the MCU, in turn, consists of the energy consumption for executing three tasks—data collection, $E_{mcu\_col}$, preprocessing, $E_{mcu\_pre}$, and MLP classification, $E_{MLP}$—as follows:

(3) $$E_{mcu}=E_{mcu\_col}+E_{mcu\_pre}+E_{mcu\_MLP}.$$

In the data collection task, the MCU operates periodically with the frequency of $f_{s}$ to read data from the sensor, switching between the active and standby states. Thus, the energy consumed by the MCU for executing the data collection task is the sum of the energy consumption in the active and standby states as follows:

(4) $$E_{mcu\_col}=t_{read}\cdot N\cdot P_{mcu\_active}+\left(t_{col}-t_{read}\cdot N\right)\cdot P_{mcu\_standby},$$

where $P_{mcu\_active}$ and $P_{mcu\_statndby}$ indicate the power consumption of the MCU in the active and standby states, respectively.

The energy consumption for executing the preprocessing, $E_{mcu\_pre}$, and the energy consumption for executing the MLP operation,$\;E_{mcu\_MLP},$ are simply estimated by:

(5) $$E_{mcu\_pre}=t_{pre}\cdot P_{mcu\_active},\;E_{mcu\_MLP}=t_{MLP}\cdot P_{mcu\_active}.$$

As mentioned, the sensor is in the active state only during data collection for time $t_{col}$, and the $E_{sensor}$ is defined as:

(6) $$E_{sensor}=t_{col}\cdot P_{sensor\_active}+\left(t_{pre}+t_{MLP}\right)P_{sensor\_idle},$$

where $P_{sensor\_active}$ and $P_{sensor\_idle}$ indicate the power consumption of the sensor in the active and standby states, respectively. Unlike the MCU, the power consumption of the sensor in the active state depends on the sampling frequency, $f_{s}$. To reflect the power consumption change by $f_{s}$, we build a power consumption model of the sensor by directly measuring the power consumption depending on $\;f_{s}$ as follows:

(7) $$P_{sensor\_active}=\alpha \cdot f_{s},$$

where α is the coefficient, which is determined as 3.56, for the flex sensor we used in the design with a 3.3 V operating voltage.

Based on Equations (3)–(7), $E_{ges}$ is finally estimated as below:

(8) $$E_{ges}=\left(t_{read}\cdot N+t_{pre}+t_{MLP}\right)\cdot P_{mcu\_active}+\left(t_{col}-t_{read}\cdot N\right)\cdot P_{mcu\_standy}+\;\alpha \cdot t_{col}\cdot f_{s}+\left(t_{pre}+t_{MLP}\right)\cdot P_{sensor\_idle}.$$

Similar to Equation (2), only $f_{s}$ and $N_{MLP}$ are major optimizable design parameters among the parameters used in Equation (8), while the other parameters such as $P_{mcu\_active}$ and $P_{mcu\_standy}$ are determined by the type of MCU device. Note that we do not consider any dynamic frequency and voltage scaling in this work, thus $P_{mcu\_active}$ and $P_{mcu\_standy}$ are constant if the same MCU devices are used in the design.

### 4.3. Energy–Accuracy Aware System-Level Design

There are numerous design choices where the energy and accuracy are trade-off relations in general. This means that maximizing recognition accuracy while simultaneously minimizing energy consumption is not easy to solve. Thus, we first define accuracy- or energy-constrained objective functions as below:

 Minimize Eges(fs,NMLP)  Subject to Acc(NMLP)≥TA or  Maximize Acc(fs,NMLP) Subject to Eges(fs)≤TE

where *T_A_* and $T_{E}$ are the given thresholds for the minimum accuracy and for the maximum energy consumption, respectively. In addition to this, we also consider a resource constraint of the devices such as the memory size of the device.

As modeled in previous sections, the sampling frequency, $f_{s}$, is a primary design factor which affects all three tasks. In general, the lower the $f_{s}$, the lower the $E_{ges}$, while lowering $f_{s}$ may negatively affect recognition accuracy. In addition to $f_{s}$, there are many other design choices as well as selecting a proper low-end device that can implement all the designs on it. For these reasons, we first discuss major system-level design choices, and then narrow down the design choices considering four types of commonly used low-end MCUs.

Using Equation (8), we can easily analyze and explore the design choices of $f_{s}$ in terms of energy consumption. However, recognition accuracy cannot be simply explored with $f_{s}$ and the other design parameters. For example, increasing $f_{s}$ may enhance recognition accuracy because it provides more information to the MLP classifier. However, improvement in accuracy is not simply proportional to $f_{s}$, and there is a saturation point. Thus, we have to find an optimal setting of $f_{s}$ through system-level design choice exploration.

In designing preprocessing filters, a simple design choice is whether each filter is adopted. We use a segmentation filter and a reshape filter for all design choices because they are indispensable while noise and normalization filters are optional. In designing a segmentation filter, determining N is tightly coupled with the setting of $f_{s}$, as shown in Equation (1), and the effects of this will be analyzed through design choice explorations as well. In terms of changing the number of sampled data from N to $N^{\prime }$ in the segmentation filter, if the difference between N and $N^{\prime }$ is larger, energy consumption in the sensor is relatively high, while the information provided to the MLP classifier is limited. Thus, we set the difference between the two numbers as close as possible by considering average $t_{col}$.

In designing a MLP classifier, finding the optimal number of parameters used in the MLP is important to find an energy–accuracy aware design. The higher the $N_{MLP}$**,** the higher the accuracy but the larger the energy consumption. Similar to $f_{s}$**,** the maximum achievable accuracy is also limited even when $N_{MLP}$ is increasing continuously. Thus, we also explore the design choices of the MLP classifier by varying $N_{MLP}$ and $f_{s}$, considering the constraint of memory space in the target device.

## 5. Evaluations

This section introduces experimental setups including the prototypes we implement to verify the energy–accuracy aware design points. Then, the results of design choice exploration and the Pareto-optimal energy–accuracy aware design points are presented with some findings and discussions.

### 5.1. Experimental Setup

**Figure 7:**
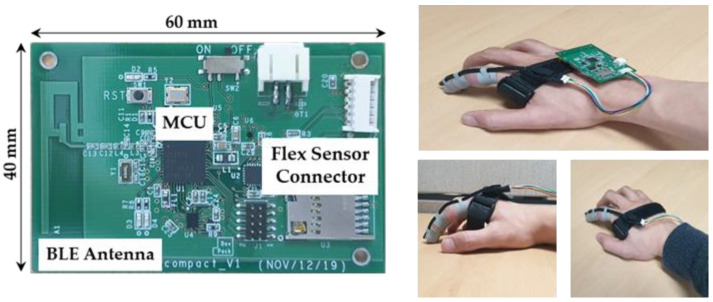
In-house prototype with CC2652R MCU.

To demonstrate the feasibility of the proposed designs, we implemented an in-house prototype tiny enough to wear on the body, as shown in Figure 7. The prototype consists of an MCU board and a flex sensor attached to the index finger. The MCU board embeds Bluetooth communication so that the recognized results can be transferred to PCs or smartphones. The flex sensor is connected through I^2^C to the MCU board. We consider four commonly used low-end MCUs for targeting low-end embedded devices. Table 2 shows the operating clock frequency, on-chip memory size, type of architecture, and power consumption of four MCUs. CC2652R shows the highest computation speed and the largest memory, including a single-precision floating point unit (FPU), while the other three MCUs have lower computation requirements and memory resources without FPUs. Note that using a hardware FPU and a different bus width of each MCU may affect the precision of floating point operation slightly. However, this issue is beyond our work because the compiler provided from each MCU handles this issue separately. In terms of power consumption in the active state, Atmega2560 has the largest active power consumption per MHz even though it is an 8-bit reduced instruction set computer (RISC) processor. In the standby state, CC2652R consumes the largest amount of power, while Atmega2560 consumes the least amount of power among four MCUs. For the flex sensor, we use a 2-axes flex sensor [14].

The prototypes are used for two purposes—data collection and design verification—through real-time gesture recognition. In data collection, the raw data collected are directly sent to the PC so that the data are used for training and for testing the MLP classifier. The prototypes are also used to provide the timing information to the energy models defined in Section 4.3. While the timing information is directly measured from the prototype board, the power consumption of the MCU is acquired from the datasheet rather than the prototype to fairly estimate only energy consumption related to gesture recognition. This means that energy estimation is not affected by the type of board implementation.

**Figure 8:**
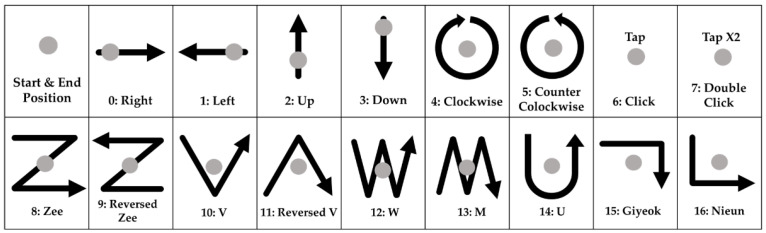
Definition of 17 finger gestures.

In total, 17 types of gestures are defined as continuous motions, as shown in Figure 8. The gray circles in the figure indicate the finger positions at the start/end of each motion. We collected a total of 5100 gestures (300 sets) from 5 users. Each set consists of 17 different gestures, and each user repeated one set of gestures 60 times. The users consist of four males and one female, with ages from 20 s to 40 s and heights from 160 to 180 cm. In order to prevent the overfitting of the trained network model and to ensure generalization ability, the collected gestures were randomly mixed among the same gestures. Then, two-thirds of collected data were used for training with the cross-validation method, while the remaining one-third of collected data were used for evaluation.

MLP training is performed in the Pytorch environment. The hyper-parameters used for trainings are 0.0075 and 500 for the learning rate and epoch, respectively. No significant performance change is observed after the epoch of 500, so the maximum epoch is fixed at 500. For comparison purposes, we build one gated recurrent unit (GRU) and two tiny ML models generated using TensorFlow and Neuton’s AutoML, which is commercially available from Google AI.

### 5.2. Results of Design Choice Exploration

**Figure 9:**
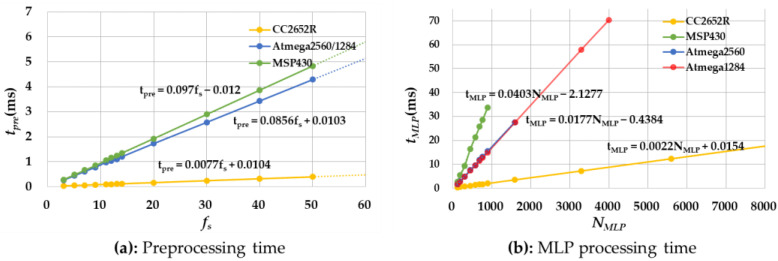
Comparison of preprocessing and MLP time.

Figure 9a shows the changes in $t_{pre}$ for four types of MCUs by increasing $f_{s}$. As expected, $t_{pre}$ is almost linearly proportional to $f_{s}$. Figure 9b shows the changes in $t_{MLP}$ by increasing $N_{MLP}$. Note that we change N into $N_{MLP}$ for simplification. Although it is not precisely linearly proportional to $N_{MLP}$, we can still use this approximate linear model based on our experiments. As shown in the graphs, the slopes are lower in the order of CC2652R, Atmega2560/1284P, and MSP430, which directly shows the computation power of each MCU.

**Figure 10:**
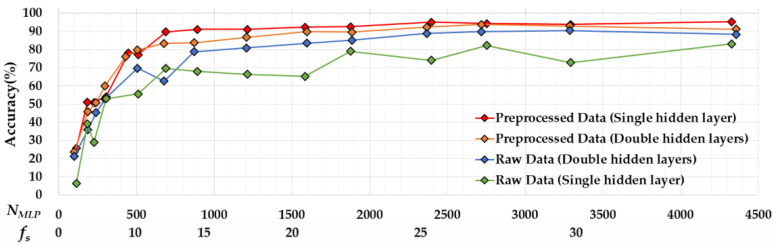
Recognition accuracy comparisons by increasing $N_{MLP}$.

Figure 10 presents the results of recognition accuracy by varying $N_{MLP}$ for the single and double hidden layers of MLPs, and also with and without preprocessing filters. In this paper, $N_{MLP}$ is calculated as:

(9) $$N_{MLP}=i\cdot h_{1}+\sum_{k=1}^{n-1}\left(h_{k}\cdot h_{k+1}\right)+h_{n}\cdot o+\sum_{k=1}^{n}h_{k}+o$$

where i and o indicate the number of nodes in the input and output layers, respectively, while $h_{k}$ is the number of nodes in the *k-th* hidden layer, and *n* is the number of hidden layers. Note that i is equal to $N^{\prime }$, which is affected by $f_{s}$. This means that $N_{MLP}$ reflects the effect of $f_{s}$ as well. For better understanding, we also mark the label of the X-axis with $\;f_{s}$.

As expected, recognition accuracy is highly correlated with $N_{MLP}$ in all four configurations. Increasing $N_{MLP}$ enhances recognition accuracy in all four configurations until $N_{MLP}$ reaches 689. However, increased accuracy starts to saturate from $N_{MLP}$ = 689 for the single hidden layer with preprocessing and from $N_{MLP}$ = 1597 for the double hidden layer with preprocessing. Clearly, applying preprocessing filters enhances accuracy for both single- and double-hidden-layer configurations. The contributions of preprocessing filters are significant especially when $N_{MLP}$ is in low regions—smaller than 900 in our experiments. In case of MSP430, which has a maximum 900 of $N_{MLP}$, the achievable maximum accuracy without a preprocessing filter is 78.7% in the single layer of MLP, while that of the one with a preprocessing filter is 91.0%.

The accuracy for the single hidden layer and double hidden layer of MLPs shows different behaviors depending on whether the preprocessing filter is applied. When preprocessing filters are not applied, the double-hidden-layer MLP shows better performance at most ranges of $N_{MLP}$. In general, it is known that using more hidden layers is useful to solve non-linear problems [25]. We observe that without preprocessing, the gesture data show more non-linearity. When processing filters are applied, the single-hidden-layer MLP shows better accuracy than the double hidden layer when $N_{MLP}$ is not sufficient. As shown in the figure, the accuracy of the single-hidden-layer MLP increases rapidly as $N_{MLP}$ increases, while that of the double-hidden-layer MLP increases relatively slowly. The accuracy of the single-hidden-layer MLP with preprocessing starts to saturate from 89.7% at $N_{MLP}$ = 689, whereas the accuracy of the double-hidden-layer MLP starts to saturate from 92.3% at $N_{MLP}$ = 1583, which uses 2.32-fold more resources. We found that applying preprocessing filters reduces the non-linearity of the data so that maximum accuracy is reached quickly to the saturation point in the single-hidden-layer MLP.

Based on comparisons of the four configurations, we conclude that the single-hidden-layer MLP with preprocessing is more suitable for devices that have limited resources.

### 5.3. Pareto-Optimal Energy–Accuracy Aware Design Points

**Figure 11:**
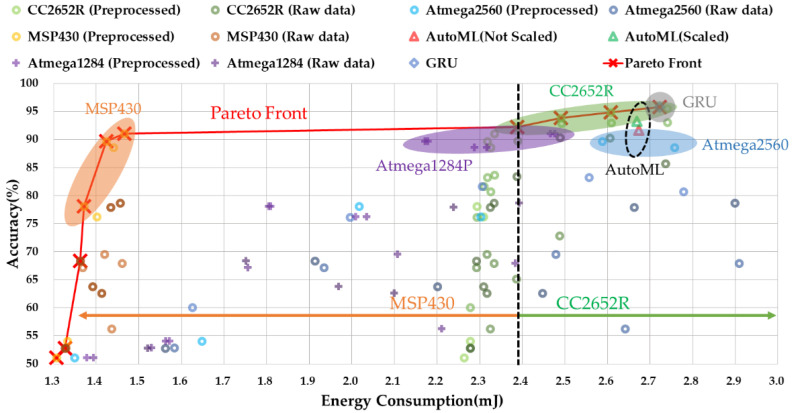
Pareto curve between energy consumption and accuracy for four types of MCUs.

We explored the design choices of the proposed finger gesture recognition system in terms of accuracy as well as the energy consumption by analyzing a total of 159 designs with varying design choices. Figure 11 shows the energy–accuracy results of each design choice as well as the Pareto-optimal designs. As shown in the figure, MSP430 and CC2652R quickly converge to peak accuracy by increasing the energy constraints. MSP430 consumes approximately half the energy compared to CC2652R while still reaching 91.0% accuracy. However, the maximum $N_{MLP}$ of MSP430 is only 900, so it cannot reach the highest achievable accuracy of 95.5%, and only CC2652R can achieve maximum accuracy even though it consumes approximately twice the energy.

Atmega2560 has the worst energy–accuracy efficiency. We found that Atmega2560 is based on an 8-bit RISC architecture, and computation requirements during the preprocessing and forward propagation operations in the MLP needs more active time of the MCU, which increases energy consumption when $\;f_{s}$ and $N_{MLP}$ increase. We observe similar energy–accuracy behaviors in Atmega1284P but with lower energy consumption than that of Atmega2560 because the active power consumption of Atmega1284P is lower than Atmega2560. Nevertheless, neither can be a Pareto-optimal.

Figure 11 also includes the energy–accuracy information of three models (one GRU and two AutoML) which are generated by a commercial platform. Due to the memory limitation, all three models are only applicable to CC2652R. The accuracy of two AutoML models are comparable to our MLP model that has 891 to 3287 parameters. However, due to the energy consumption, those models cannot be selected as Pareto optimal. The GRU model shows slightly better accuracy than our design, with similar energy consumption. Thus, it can be selected as a Pareto-optimal solution if CC2562R or higher MCU is used for the target device. However, this GRU model cannot be a solution if the user wants to implement it on a low-end MCU such as MSP430 or lower.

Table 3 shows the design choices of each Pareto Front in detail. If the accuracy is given as a design constraint, MSP430 can be used if the given accuracy is under 91.0% while CC2652R MCU can be used over 91.0% of constraints. When energy consumption is a major constraint of the design, MSP430 is mostly used if the budget of the energy is under 2.39 mJ per gesture while CC2652R is used if the energy budget is over 2.39 mJ. ATmega2560/1284P can still be considered as a target MCU if the users want to reuse the hardware and software they have already developed. In this case, the results of our exploration could be useful as well.

**Figure 12:**
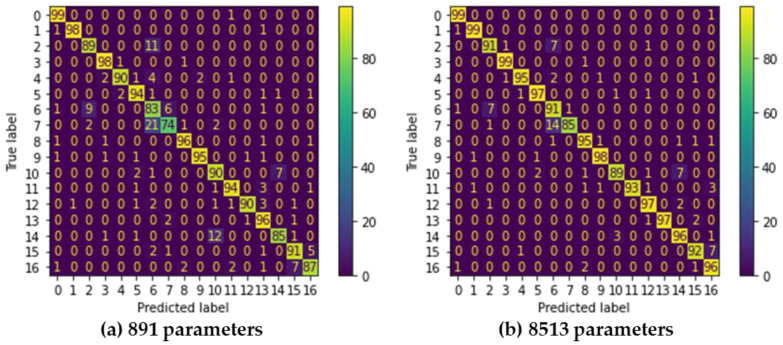
Confusion matrices of two Pareto Fronts.

**Figure 13:**
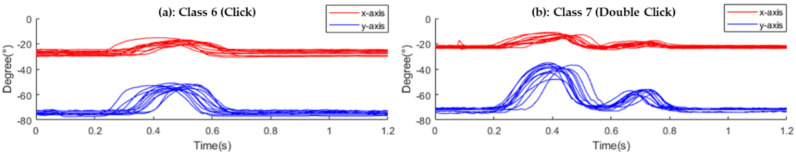
Raw data collected on gestures 6 and 7 from the flex sensor.

A confusion matrix is useful for analyzing the patterns of mispredictions. Figure 12a shows the confusion matrix of a model using 891 parameters with an accuracy of 91.0% and an energy consumption of 1.47 mJ when using a MSP430. In this design, 21.0% of “Double Click” gestures (class 7) are mispredicted as “Click” gestures (class 6). As defined in Figure 8, “Click” moves the finger up and down once, while “Double Click” moves the finger up and down in the same way but twice. Figure 13 shows the raw data collected on two gestures directly from the sensors. As shown in the figures, the patterns of the two gestures are similar, thus the model with 891 parameters is not enough to distinguish them clearly.

Figure 12b shows the confusion matrix of the classifier using 8513 parameters, which is 9.55-fold greater than using 891 parameters. This design achieves 95.5% accuracy with an energy consumption of 2.74 mJ when using CC2652R. Nevertheless, 14.0% of “Double Click” gestures (class 7) are mispredicted as “Click” gestures (class 6). This may indicate that simple MLP may not be a perfect solution to completely distinguish these two gestures. Although this design shows a lower number of mispredictions than the design with 891 parameters, energy consumption is increased by 1.86 fold, while improvement in accuracy is only 4.4%. In addition, this design cannot be implemented in MSP430 because of memory shortage. Table 4 summarizes and compares this work with existing hand/finger gesture recognition designs, in terms of the sensors, classification models with size information, the number of recognized classes, accuracy, and implementation. We do not directly compare recognition accuracy because the target applications, type of sensor, the number of recognized classes, and the dataset used for training and testing are different in each work. As shown in the table, most studies only provide the design and performance analysis without details on implementation issues. The work in [3,7] tried to reduce model size and can be implemented in MCU devices, but not on low-end MCUs with only a few tens of KB memory and low computing resources. The work in [8] was implemented on an Arduino Due board. However, the Arduino board only collects and preprocesses the collected data while classifications are performed on Field Programmable Gate Arrays (FPGAs). Most of all, none of the existing studies considers energy–accuracy design choices, which is very important for designing wearable IoT devices.

## 6. Conclusions

In this paper, we implemented a finger gesture recognition system based on a light-weight MLP-based classifier using a low-end MCU and a 2-axes flex sensor. In order to find energy–accuracy aware design points, we first designed a full process of finger gesture recognition and its system-level performance and energy models. Then, we analyzed system-level design issues including sensor operating frequency and the size of the MLP classifier. Finally, we explored the numerous design choices based on accuracy and energy constraints. Considering four commonly used MCUs, a total of 159 design points were determined according to the configuration of the sensor operating frequency, the presence of preprocessing filters, and the size of the MLP classifier. As a result of Pareto Fronts, the proposed design achieved up to 95.5% accuracy with an energy consumption of 2.74 mJ, which shows up to 10% higher accuracy than previous studies [26] with similar low-end MCUs. Collectively, this study details how to achieve energy–accuracy aware design points under given energy or accuracy constraints.

In this work, we do not address the effect of using AI accelerators such as digital signal processors (DSPs), FPGAs or application-specific integrated chips (ASICs). Since these accelerators will greatly affect performance as well as energy efficiency, considering these components will be our future work to find energy–accuracy aware design choices for wearable IoT devices.

## Figures and Tables

**Table 1:** Description of the parameters used in the model.

Definition	Description
N	Number of sampled data per gesture to be recognized
$N_{MLP}$	Number of parameters used in the MLP classifier
$f_{s}$	Sensor frequency (sample rate)
$t_{ges}$	Time taken per gesture recognition $=t_{col}+t_{pre}+t_{MLP}$
$t_{read}$	Time taken to read one sample from the sensor 269 us (including time to wakeup, I^2^C transfer, time to sleep)
$t_{pre}$	Time taken to perform preprocessing Depends on $f_{s}$
$t_{MLP}$	Time taken to perform the MLP evaluation Depends on # of parameters in the $f_{s}$
$t_{col}$	Time taken to collect data $=\left(t_{read}+t_{standby}\right)\times N$

**Table 2:** Characteristics of the low-end MCUs used in this work.

MCU	Clock Frequency (MHz)	On-Chip Memory (KB)	$\mathrm{Max}.\;N_{MLP}$	Architecture	Active Current (mA/MHz)	Standby Current (uA)
CC2652R	48	80	18,100	CortexM4F 32 bit RISC	0.07	675
Atmega2560	16	8	1972	AVR 8 bit RISC	2.3	170
Atmega1284P	16	16	3960	AVR 8 bit RISC with picoPower	0.86	210
MSP430	16	4	900	16 bit RISC	0.13	420

**Table 3:** Details of the Pareto Front design choices.

MCU Type	Sample Rate	*N_MLP_*	Memory Size (Byte)	MLP Layers	Accuracy (%)	*E_ges_* (mJ)
MSP430	5	185	740	10 × 6 × 17	51.1	1.31
	7	297	1188	14 × 7 × 7 × 17	60.1	1.33
	9	449	1796	18 × 12 × 17	78.1	1.36
	11	589	2356	22 × 11 × 11 × 17	81.7	1.37
	12	689	2756	24 × 16 × 17	89.7	1.42
	14	891	3564	28 × 19 × 17	91.0	1.47
CC2652R	20	1583	6332	40 × 27 × 17	92.3	2.39
	30	3287	13,148	60 × 30 × 30 × 17	92.9	2.49
	40	5603	22,412	80 × 57 × 17	94.8	2.61
	50	7787	31,148	GRU	95.8	2.72

**Table 4:** Comparisons of existing hand gesture recognition studies.

	[1]	[3]	[5]	[6]	[7]	[8]	[13]	[23]	[26]	This Work
Used sensors	Camera	EMG (Myo)	Depth camera	Optical and IMU	Flex Sensor	IMU	Pressure, flex, gyro, IMU, etc.	Accelerometer	Flex sensor	2-axes flex sensor
Models (num. of parmas or mem. size)	CNN + RNN (N/A)	CNN (34 K)	Custom (600 MB)	HMM (N/A)	GRU + MAP (50 K~)	RCE (274.3 Kb)	LSTM (N/A)	RNN (69 K)	AL ^1^ (N/A)	MLP (185~8513)
Classes	4	7	124	26	4	10	31	8	4	17
Accuracy (%)	96.4	98.8	91.9	98.1	97.3	98.6	90.0	88.6	88.3	95.5
Implementation	N/A	N/A	Inter i5, GPU (GTX750)	N/A	Raspberry Pi 3	Arduino + FPGA	N/A	N/A	N/A	CC2652R, Atmega, MSP430

^1^ AL: adversarial learning.
